# An innovative CASC training redesign – ‘experience of virtual mock CASC exam’

**DOI:** 10.1192/bjo.2021.428

**Published:** 2021-06-18

**Authors:** Jennifer Rankin, Jessica Foster, Omer Minhas

**Affiliations:** 1 Aneurin Bevan UHB; 2 Cardiff & Vale UHB; 3 Tonteg Hospital

## Abstract

**Aims:**

Cardiff CASC Training (CCT) has provided structured and formal training for the CASC exam for Wales trainees since 2012, in conjunction with Wales Deanery. For the past 8 years CCT has delivered face-to-face mock CASC exams and received excellent feedback from candidates and examiners, in addition to an extremely positive outcome of improvement in CASC pass rate for candidates.

Due to the current COVID-19 pandemic restrictions delivery of the mock CASC examination had to be adapted with the aim of running it remotely via an online platform.

**Method:**

The examinations were run online via Zoom due to its ease of use, including the ability to screen share candidate instructions and assign participants in to breakout rooms. One lead exam coordinator manually rotated candidates around the circuit of 16 stations.

**Result:**

A total of 16 candidates sat the mock exam over two separate sittings. Written feedback was obtained from candidates and examiners. Limitations identified during the initial sitting included high logistical workload for the lead exam co-ordinator and Zoom not being supported by all hospital computer internet browsers, these issues were addressed prior to the second sitting. Feedback from candidates regarding the overall experience of the online exams ranged from 'extremely effective’ to ‘very effective’, this is in line with feedback obtained following previous face-to-face mock exam events CCT has run.

**Conclusion:**

Although online learning may feel very different to the face-to-face interactions we are all used to we are in an era where adaption is necessary. These online mock CASC examinations have been a success and are also in keeping with how the real CASC examination is currently being run by the Royal College of Psychiatrists. CCT are running a further online mock examination to support the next cohort of candidates through their CASC exam in this particularly challenging time.

